# The Role of Abutment Preparation Strategies in Optimizing the Bond Strength of Resin-Bonded Fixed Partial Dentures: An In Vitro Study

**DOI:** 10.7759/cureus.92929

**Published:** 2025-09-22

**Authors:** Ameena Abdussalam, Prasad Rajagopal, Jyothsna M Karumuthil

**Affiliations:** 1 Department of Prosthodontics, Educare Institute of Dental Sciences, Malappuram, IND; 2 Department of Prosthodontics, Kerala University of Health Sciences, Thrissur, IND

**Keywords:** abutment, design, fixed partial dentures, micromechanical, preparation, resin bonded

## Abstract

Introduction

Prosthodontics has increasingly embraced minimally invasive techniques, with resin-bonded fixed partial dentures (RBFPDs) offering a conservative solution for anterior tooth replacement. The success of RBFPDs depends on the durability of the bond between the retainer and abutment, which might be influenced by tooth preparation design. This study aimed to compare the shear bond strength of RBFPDs across four abutment preparation designs to identify the optimal configurations for enhanced retention.

Materials and methods

This in vitro study was conducted at the Educare Institute of Dental Sciences in Malappuram, India. Forty acrylic samples, each with one maxillary central incisor and one canine, were divided into four groups (n = 10): Group A (control: 0.5 mm lingual reduction with chamfer), Group B (tram-line: three grooves, two lingual, one facio-proximal), Group C (lingual rest seat with one distolingual groove), and Group D (two vertical parallel grooves). Acrylic blocks were fabricated using standardized wax molds, and tooth preparation was performed using a diamond bur. Metal dies and cobalt-chromium retainers (Wironit, Bego, Bremen, Germany) were cast, abraded with 50 µm aluminum oxide, and cemented with a resin cement (Dentsply Sirona, Charlotte, NC, USA). The shear bond strength was tested using a universal testing machine (Instron 5944, Instron Corporation, Norwood, MA, USA) at 1 mm/min, with values recorded in Newtons (N). Statistical analysis was performed using one-way ANOVA and the post hoc Tukey test (p < 0.05).

Results

One-way ANOVA revealed significant differences in bond strength (p < 0.001). Group B exhibited the highest mean bond strength (370.49 ± 27.24 N), followed by Group D (273.19 ± 23.03 N), with Groups A (147.44 ± 9.46 N) and C (156.18 ± 9.66 N) showing lower, comparable values. Post hoc tests confirmed Group B’s superiority (p < 0.001), with no significant difference between Groups A and C (p = 0.055).

Conclusions

The tram-line preparation significantly enhanced RBFPD retention, followed by the dual-groove design, supporting their use in conservative prosthodontics. Further in vivo studies are required to validate its clinical applicability.

## Introduction

The field of prosthodontics has witnessed remarkable advancements over the decades, with a growing emphasis on conservation in the 21st century, mirroring dentistry’s broader transition toward minimally invasive procedures [1]. As this approach permeated various dental disciplines, it laid the groundwork for innovations in fixed partial prosthodontics, particularly the development of resin-bonded fixed partial dentures (RBFPDs), which balance functional restoration with minimal tissue alteration [2]. The adoption of RBFPDs signifies a paradigm shift in the prosthetic management of missing teeth, offering a minimally invasive, partially reversible, affordable, and time-efficient alternative to traditional methods. Their appeal is especially pronounced in younger patients, where preserving the natural tooth structure is paramount [3,4].

In the historical evolution of RBFPDs, the core challenge lies not in resin-enamel bonding but in achieving reliable adhesion between the metal frameworks and resin [5]. Various techniques have been devised to overcome this problem, including micromechanical interlocking, macromechanical retention, and advanced adhesive mechanisms [6,7]. Rochette [6] pioneered the application of RBFPDs in 1973 for periodontally compromised anterior teeth, using perforated cast metal retainers for mechanical retention via resin interdigitation. This was expanded in 1977 by Howe and Denehy [7], who adapted this technique for interim partial dentures. Subsequent advancements in refined materials and designs: cast-perforated (Rochette) bridges rely on perforations for mechanical hold, Virginia bridges incorporate macroscopic undercuts for enhanced retention, and etched-cast (Maryland) bridges employ electrolytic etching to create micromechanical surface topography for superior resin adhesion [8].

Early RBFPDs had high failure rates due to debonding, but improvements in tooth preparation designs, intaglio surface treatments, and adhesive resins have elevated survival rates, ranging from 59% to 100%; a systematic review reported a medium-term average of 87.7% [9]. Debonding remains the primary failure mode, followed by caries and retainer fractures; however, RBFPDs have gained favor owing to their conservative nature. However, research on the influence of abutment preparation on retention is limited. Enhancing retainer anchorage through modifications, such as proximal grooves for defined insertion paths and resistance to displacement, cingulum rests, occlusal rest seats, and lingual clearances, mitigates occlusal stresses and bolsters bond durability [10,11]. Currently, RBFPD applications are confined mainly to anterior regions with low forces; however, optimized bonding can expand their utility. Prosthetic longevity depends on the preparation geometry, material properties, adhesive integrity, retainer coverage, and clinical expertise, all of which reduce debonding risk [11].

This in vitro experimental study aimed to evaluate and compare the retention of RBFPDs across four different abutment modification designs on anterior teeth. Specifically, we assessed the shear bond strength in preparations featuring a 0.5 mm lingual reduction with a chamfer finish line as the control group and the shear bond strength in various modified tooth preparations and conducted a comparative analysis of shear bond strengths across all groups to highlight the role of abutment design in enhancing prosthesis survival.

## Materials and methods

This in vitro experimental study was designed in a controlled laboratory setting to ensure standardized conditions. The study was conducted in the Department of Prosthodontics at the Educare Institute of Dental Sciences in Malappuram, India. As the study exclusively utilized synthetic acrylic samples and Ivorine teeth, it did not involve human or animal subjects; therefore, ethical approval was not required.

Forty specimens, divided equally into four groups (n = 10 per group), were evaluated to assess the impact of abutment modifications on retention. The sample size was determined using G*Power software version 3.1.9.4 (Heinrich Heine University Düsseldorf, Düsseldorf, Germany) with an alpha error probability of 0.05, power (1-β) of 0.80, and an effect size of 0.57 derived from a prior study by Saad et al. [10], resulting in a requirement of 10 samples per group.

To fabricate acrylic samples, eight ivorine teeth, four maxillary central incisors, and four maxillary canines (Columbia Dentoform, Dentalez, Lancaster, PA, USA) were mounted on acrylic resin blocks (Premium Denture Acrylic, Wheeling, IL, USA). Rectangular wax blocks measuring 1 × 2 × 3 cm were created using modeling wax (Kerr Modeling Wax, Kerr Corp., CA, USA), and silicone indices were made with polyvinyl siloxane impression material (Aquasil Ultra, Dentsply Sirona, Charlotte, NC, USA). Each block featured one central incisor and one canine, with a standardized space equivalent to a maxillary lateral incisor, maintained by a removable wax block matching the mesiodistal width of the lateral incisor. Four blocks were prepared and designated as Group A (control), Group B (tram-line), Group C (lingual rest with distolingual groove), and Group D (two proximal grooves, mesial and distal).

**Figure 1:**
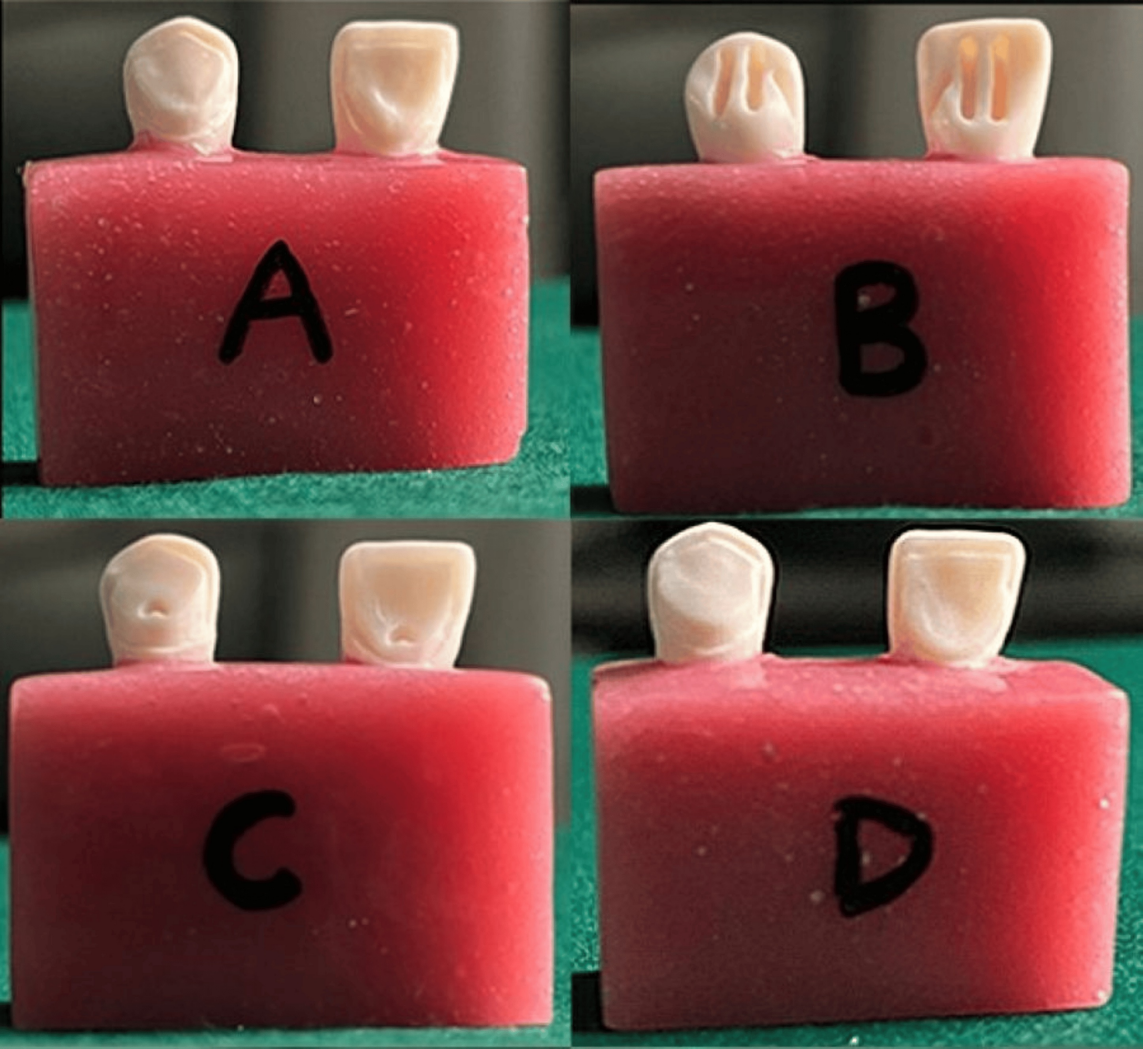
Tooth preparation designs (A) Group A as control group with lingual reduction and chamfer finish line, (B) Group B where tram-line was prepared with lingual and facio-proximal grooves, (C) Group C where cingulum rest seat was prepared with distolingual line angle groove, and (D) Group D where two vertical parallel grooves at mesiolingual and distolingual line angles were prepared. Original images of samples from the study.

Tooth preparation was performed using a round-ended tapered diamond bur (Premier Solo Diamond, Premier Dental Products, PA, USA) attached to an air turbine handpiece (Bien-Air Bora, Bien-Air Dental, Switzerland). For Group A (control), lingual surfaces were reduced by approximately 0.5 mm, and a chamfer finish line was shaped 1 mm coronal to the cementoenamel junction. Group B (tram-line) featured three grooves: two shallow grooves (1 mm depth, 5 mm length, and 0.75 mm width) on the lingual surface and one groove (1 mm depth, 2 mm length, and 0.75 mm width) at the facio-proximal line angle. Group C included a lingual rest seat in the cingulum with tapered walls, a flattened base, and a distolingual line angle groove (2 mm length, 1 mm depth, and 1 mm width). Group D had two vertical parallel grooves (2 mm length, 1 mm depth, and 1 mm width each) at the mesiolingual and distolingual line angles, aligned with the prosthesis insertion path (Figure 1).

**Figure 2:**
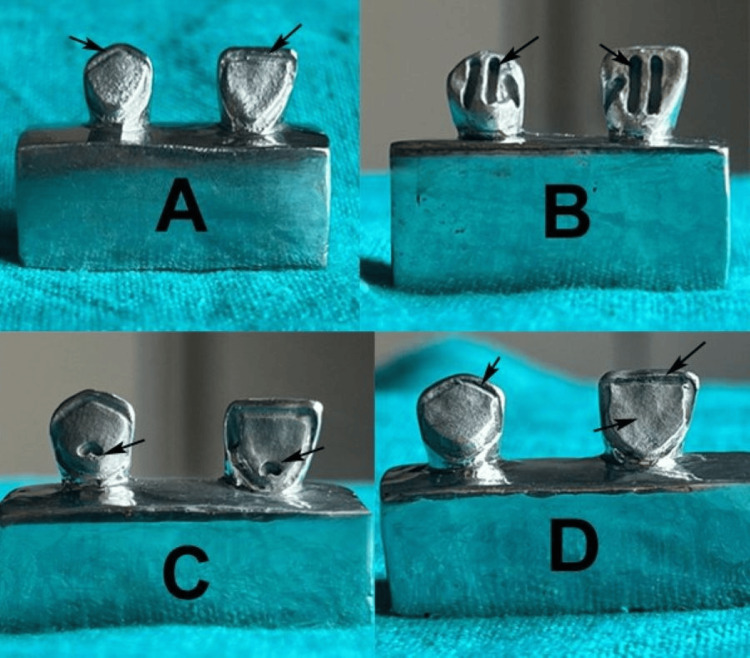
Fabricated metal dies (A) Group A as control group with lingual reduction and chamfer finish line, (B) Group B where tram-line was prepared with lingual and facio-proximal grooves, (C) Group C where cingulum rest seat was prepared with distolingual line angle groove, and (D) Group D where two vertical parallel grooves at mesiolingual and distolingual line angles were prepared. Original images of samples from the study.

Metal dies were fabricated by taking impressions of the prepared acrylic blocks using custom trays and polyvinyl siloxane material. Wax patterns were created via the direct method by pouring molten inlay pattern wax (Kerr Inlay Wax, Kerr Corp.) into the impressions. After setting, the patterns were retrieved, sprued, and sprayed with surfactant (Beta-Cast Surfactant, TAUB Products, NJ, USA). Patterns were invested in phosphate-bonded investment material (Ceramigold, Whip Mix Corp., KY, USA), subjected to wax burnout at 950°C in a furnace, and cast with a cobalt-chromium alloy (Wironit, Bego, Bremen, Germany) using centrifugal force. Sprues were removed, and the dies were cleaned, finished, and polished. This process was repeated for all the groups (Figure 2).

**Figure 3:**
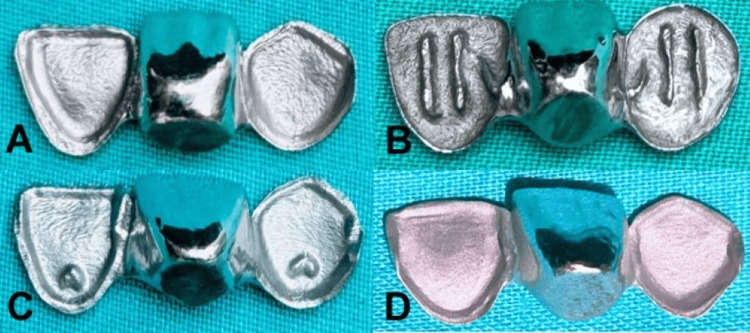
RBFPD retainers fabricated for each preparation design: (A) control retainer, (B) tram-line retainer, (C) cingulum rest seat retainer, and (D) vertical groove retainer. RBFPD, resin-bonded fixed partial denture Original images of samples from the study.

RBFPD metal retainers were fabricated by building wax patterns on each metal die using inlay pattern wax. Patterns were sprued, invested in phosphate-bonded material, subjected to burnout, and cast with a cobalt-chromium alloy. After casting, the retainers were cleaned, finished, and uniformly polished. For cementation, the intaglio surfaces of the retainers were abraded with 50 µm aluminum oxide (Zest Dental Sol., Carlsbad, CA, USA) at a pressure of 5 bar for 30 seconds, cleaned in acetone for five minutes, and air-dried. The prosthesis fit was verified, and cementation was performed with a resin cement (Calibra dual-cure, Dentsply Sirona) following the manufacturer’s protocols (Figure 3).

Shear bond strength testing was conducted on all 40 samples using a universal testing machine (Instron 5944, Instron Corporation, Norwood, MA, USA) set at a crosshead speed of 1 mm/min. The specimens were mounted at an angle of 30° in a custom-fabricated jig, and a chisel applicator applied a central load until retainer debonding occurred. The shear bond strength values were recorded in Newtons (N). After debonding, the residual cement was removed using an explorer, and the surfaces were reconditioned via sandblasting. Calibration of the testing machine was performed per the ISO 7500-1 standards, with load cell verification using standard weights (accuracy ±0.5%) and crosshead speed confirmed with a digital tachometer. A pilot test on five preliminary samples yielded an intraclass correlation coefficient of 0.92, indicating a high reliability. All preparations and measurements were performed by a single operator, blinded to group allocation, with groove dimensions verified using digital calipers (Mitutoyo, Kawasaki, Japan) with 0.01 mm resolution.

Statistical analysis

Statistical analyses were performed using IBM SPSS Statistics for Windows, Version 26.0 (Released 2018; IBM Corp., Armonk, NY, USA). The normality of the continuous bond strength data was confirmed using the Shapiro-Wilk test. Consequently, parametric tests were conducted. One-way ANOVA followed by a post hoc Tukey test was used to compare the mean shear bond strength among groups and for pairwise comparisons. Statistical significance was set at p < 0.05.

## Results

A one-way ANOVA revealed a statistically significant difference in the mean shear bond strength between the groups (F = 308.61, p < 0.001). Group B demonstrated the highest mean shear bond strength (370.49 ± 27.24 N), followed by Group D (273.19 ± 23.03 N). Groups A (147.44 ± 9.46 N) and C (156.18 ± 9.66 N) recorded the lowest values. The highly significant p-value indicates that the observed variation in means is very unlikely to be due to random chance, confirming that shear bond strength is profoundly influenced by the group type or treatment applied (Table 1).

**Table 1:** Comparison of mean shear bond strength in newtons (N) of RBFPDs across abutment preparation groups. ^*^ p = 0.001 indicates statistical significance for one-way ANOVA comparing shear bond strength across groups. Sample distribution is shown as frequency (N) and percentage; shear bond strength is reported as mean and SD. RBFPD, resin-bonded fixed partial denture

Groups	N (%)	Mean	SD	Minimum	Maximum	F value	p-Value
Group A (control)	10 (25%)	147.44	9.46	138.18	168.82	308.61	0.001^*^
Group B (tram-line)	10 (25%)	370.49	27.24	336.81	411.6		
Group C (lingual rest with distolingual groove)	10 (25%)	156.18	9.66	133.77	164.95		
Group D (two proximal grooves - mesial and distal)	10 (25%)	273.19	23.03	231.24	293.34		

Post hoc Tukey test revealed significant pairwise differences in shear bond strength. The mean shear bond strength in Group B was significantly higher than that in all other groups (p < 0.001). Conversely, the difference between Groups A and C was not significant (p = 0.055). The mean shear bond strength of Group D was significantly greater than that of both Group A and Group C (p < 0.001), but significantly lower than that of Group B (p < 0.001). This indicates a distinct performance hierarchy: Group B was superior; Group D was intermediate; and Groups A and C were statistically equivalent and represented the lowest shear bond strengths (Table 2).

**Table 2:** Pairwise comparisons of mean shear bond strength differences in newtons (N) among Group A (control), Group B (tram-line), Group C (lingual rest with distolingual groove), and Group D (two proximal grooves - mesial and distal) using the post hoc Tukey test. ^*^ p = 0.001 indicates statistical significance for pairwise comparisons using the Tukey test, adjusted for multiple comparisons.

Pairwise groups		Mean difference (N)	t-Value	p-Value
Group A	Group B	223.04	24.46	0.001^*^
	Group C	8.74	2.04	0.055
	Group D	125.75	15.97	0.001^*^
Group B	Group C	214.3	23.44	0.001^*^
	Group D	97.29	8.62	0.001^*^
Group C	Group D	117.01	14.81	0.001^*^

## Discussion

This in vitro study investigated the shear bond strength of anterior RBFPDs across four distinct abutment preparation designs and revealed significant differences influenced by the presence and configuration of mechanical retention features. The tram-line preparation (Group B), incorporating three grooves (two shallow lingual and one facio-proximal), demonstrated superior bond strength compared to the dual vertical groove design (Group D), which featured mesiolingual and distolingual grooves aligned with the insertion path. In contrast, the control group, with only lingual reduction and a chamfer finish line, and Group C, with a lingual rest seat and a single distolingual groove, exhibited lower and comparable bond strength. Statistical analysis confirmed the significant impact of the preparation design on bond strength, with post hoc tests establishing a clear performance hierarchy: tram-line superior to dual grooves, which outperformed the control and single-groove designs. These findings underscore the critical role of groove-based mechanical retention in enhancing RBFPD durability, aligning with prosthodontics’ shift toward conservative yet robust restorative solutions. el-Mowafy and Rubo [12] concluded that the retention of an RBFPD is not related to the type of resin cement or the surface area of castings; rather, it is related to the mechanical interlocking of the castings with the retentive-slot resin composite restorations.

The exceptional performance of Group B can be attributed to its three-groove configuration, which increases the surface area for micromechanical interlocking between the cobalt-chromium retainer and resin cement. This design is likely to distribute shear stresses more effectively, reducing the force concentration at the bond interface and minimizing adhesive or cohesive failures. Similarly, Saad et al. [10] found that longer grooves in maxillary anterior RBFPDs enhanced retention by improving resin interdigitation and resistance to vertical forces. It is hypothesized that retention grooves safeguard the adhesive layer of resin-bonded appliances against delamination forces, consequently enhancing their retentive strength in clinical contexts. Hansson and Bergström [13] further supported this finding, reporting that multiple grooves improved bond durability by enhancing resin penetration and counteracting torsional stresses, which is consistent with the multidirectional resistance of the tram-line design in this study. In contrast, Brune et al. [14] concluded that the number of retention grooves and the size of the adhesive area did not significantly affect the failure load of the RBFPDs.

The intermediate shear bond strength of Group D reflects the benefits of two parallel vertical grooves, which provide macromechanical retention against lingual and cervical displacement while preserving enamel. Nair et al. [15] noted that the placement of dual parallel grooves increased retentive forces compared to single-groove or grooveless designs because of enhanced parallelism and interlocking, while Emara et al. [16] found that vertical proximal grooves increased for maxillary molars by creating defined insertion paths that resist occlusal forces. However, with fewer grooves than in Group B, Group D’s mechanical advantage was less pronounced, explaining its position in the performance hierarchy.

The lower shear bond strengths in Groups A and C highlight the limitations of minimal mechanical enhancements. Group A’s reliance on chemical adhesion to the enamel-like ivorine surface, without auxiliary retention, mirrors the early RBFPD failures reported in the literature, where debonding was prevalent due to insufficient mechanical support [15]. Group C’s lingual rest seat and single groove provided some anti-rotational stability but failed to significantly enhance shear resistance, likely due to limited surface area augmentation. Kern and Strub [17] concluded that cingulum rest alone contributes minimally to bond strength, whereas multiple groove systems offer substantial improvements. Miettinen and Millar’s [18] review further noted that preparations lacking multiple retentive elements are prone to higher debonding rates, corroborating the vulnerabilities observed in Groups A and C.

The clinical implications of our findings are significant. The superior shear bond strength of the tram-line design suggests that it could enhance RBFPD longevity, potentially expanding its use to regions with moderate occlusal forces, such as posterior areas, while maintaining a conservative approach ideal for younger patients. The dual-groove design offers a viable alternative where minimal preparation is preferred, balancing retention and enamel preservation. Clinicians should prioritize groove-based preparations to mitigate debonding risks and tailor designs for patient-specific occlusal and aesthetic needs. However, this study has some limitations that must be acknowledged. The use of ivorine teeth and metal dies may not fully replicate the viscoelastic properties of natural enamel and dentin, potentially affecting the bond strength estimates. The absence of cyclic loading or thermocycling, which simulates oral conditions, limits clinical extrapolation. Additionally, the testing was conducted under controlled conditions, excluding variables such as saliva or occlusal dynamics. Future research should incorporate in vivo studies or fatigue testing to validate the clinical efficacy of these designs and explore their performance in dynamic oral environments.

## Conclusions

This in vitro study concluded that the configuration of mechanical retention features significantly influenced prosthetic retention. The tram-line preparation, incorporating three grooves, demonstrated the highest bond strength, followed by the dual vertical groove design, while the control preparation with lingual reduction and chamfer and the lingual rest seat with a single groove exhibited comparable lower bond strengths. These findings highlight the critical role of multiple groove-based designs in enhancing the micromechanical interlocking and stress distribution, thereby reducing debonding risks. The superior performance of the tram-line preparation suggests its potential to improve RBFPD longevity, particularly for anterior restorations, while the dual-groove design offers a balanced alternative for conservative applications.
