# Correction: The Role of Abutment Preparation Strategies in Optimizing the Bond Strength of Resin-Bonded Fixed Partial Dentures: An In Vitro Study

**DOI:** 10.7759/cureus.c454

**Published:** 2026-06-22

**Authors:** Ameena Abdussalam, Prasad Rajagopal, Jyothsna M Karumuthil

**Affiliations:** 1 Department of Prosthodontics, Educare Institute of Dental Sciences, Malappuram, IND; 2 Department of Prosthodontics, Kerala University of Health Sciences, Thrissur, IND

This article has been corrected to update Dr. Ameena Abdussalam’s affiliations to: Department of Prosthodontics, Educare Institute of Dental Sciences, Malappuram, IN and Department of Prosthodontics, Kerala University of Health Sciences, Thrissur, IN.

